# Supplementary material for: Targeting human Acyl-CoA:cholesterol acyltransferase as a dual viral and T cell metabolic checkpoint
Source: Nat Commun. 2021 May 14;12:2814. doi: 10.1038/s41467-021-22967-7 (PMC8121939; doi:10.1038/s41467-021-22967-7)
Supplement: Supplementary file 1 — Supplementary Information [file 41467_2021_22967_MOESM1_ESM.pdf]

Targeting human Acyl-CoA:cholesterol acyltransferase as a dual viral and T-cell metabolic checkpoint

Nathalie M Schmidt, Peter A C Wing, Mariana O Diniz, Laura J Pallett, Leo Swadling, James M Harris, Alice R Burton, Anna Jeffery-Smith, Nekisa Zakeri, Oliver E Amin, Stephanie Kucykowicz, Mirjam H Heemskerk, Brian Davidson, Tim Meyer, Joe Grove, Hans J Stauss, Ines Pineda-Torra, Clare Jolly, Elizabeth C Jury, Jane A McKeating, Mala K Maini

Supplementary Figure 1

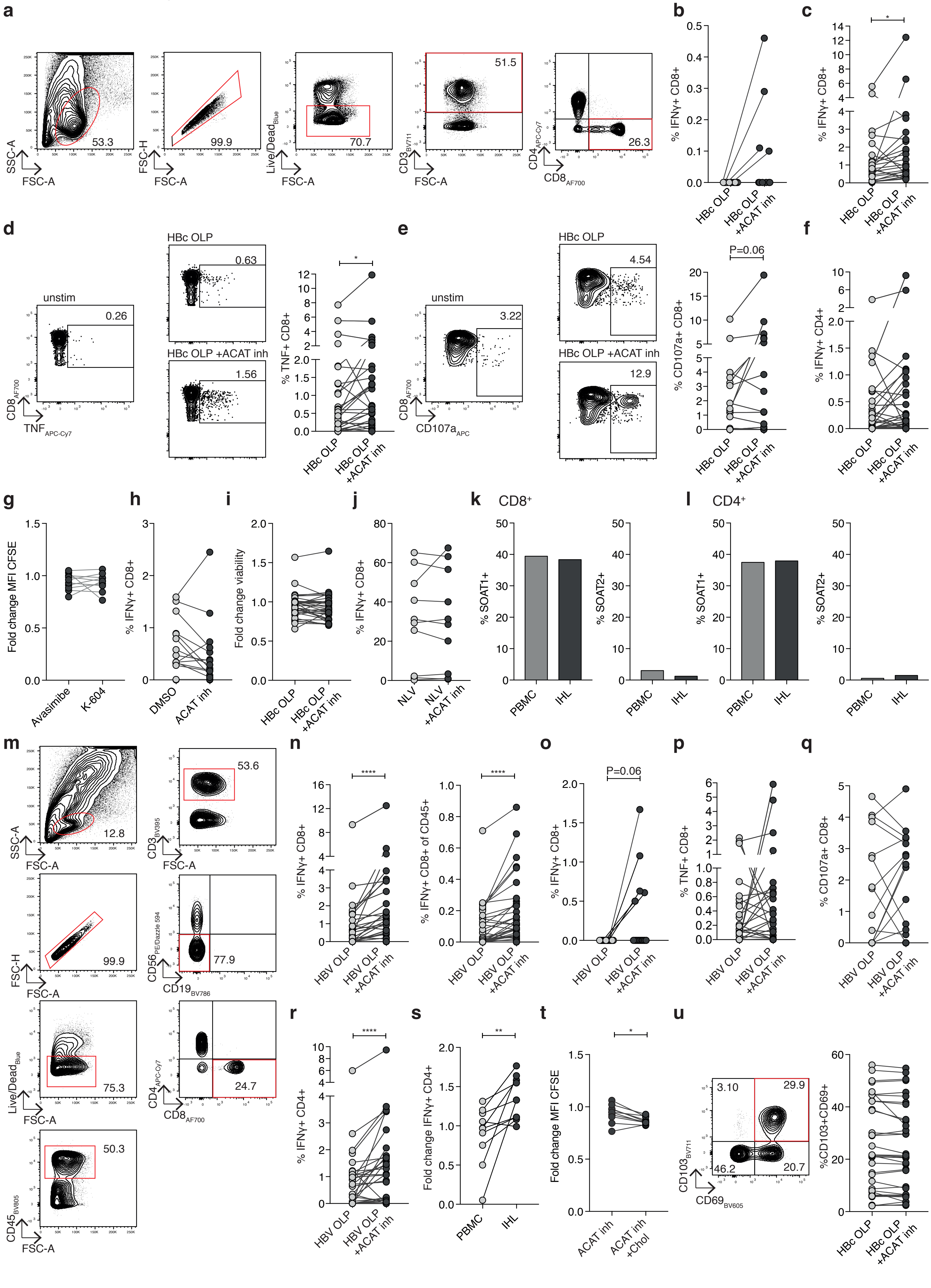

Supplementary Figure 1 ACAT inhibition enhances peripheral and intrahepatic HBV-specific T-cells.

(**a-f**) PBMC from patients with CHB were stimulated with HBc OLP ±ACAT inhibition (Avasimibe or equivalent concentration of DMSO for 8d) and cytokine production was detected via flow cytometry. The cytokine production in wells without peptide stimulation was subtracted to determine HBV-specific cytokine production in summary data. (**a**) Representative flow cytometry plot of gating strategy for PBMC: lymphocytes, single cells, live cells, $CD3^+$, $CD4^-/CD8^+$. (**b**) *De novo* IFNγ production of $CD8^+$T-cells ±ACAT inhibition in individuals with no detectable baseline response to HBc OLP (n=10). (**c**) Summary data for HBV-specific IFNγ production by $CD8^+$T-cells ±ACAT inhibition (n=34). (**d+e**) Representative flow cytometry plot and summary data for HBV-specific TNF production (**d**, n=30) and CD107a mobilisation (**e**, n=17) by $CD8^+$T-cells ±ACAT inhibition. (**f**) HBV-specific IFNγ production by $CD4^+$T-cells ±ACAT inhibition. (**g**) Comparison of proliferation of $IFN\gamma^+$ $CD8^+$T-cells by CFSE dilution after stimulation with aCD3/aCD28 +ACAT inhibition (Avasimibe versus K-604; n=10 for 4d). (**h**) IFNγ production by unstimulated $CD8^+$T-cells ±ACAT inhibition (Avasimibe or DMSO for 8d; n=15). (**i**) Fold change viability of PBMC stimulated with HBc OLP ±ACAT inhibition (Avasimibe or DMSO for 8d) normalized to unstimulated control (n=34). (**j**) PBMC from patients with CHB (n=10) were stimulated with CMV-derived peptide (NLV) ±ACAT inhibition (Avasimibe or DMSO for 8d) and NLV-specific IFNγ production was detected via flow cytometry. The IFNγ production in wells without peptide stimulation was subtracted to determine NLV-specific IFNγ production. (**k+l**) $\%CD8^+$ (**k**) or $\%CD4^+$ (**l**) T-cells with detectable SOAT1 (ACAT1) or SOAT2 (ACAT2) gene transcripts by single-cell (sc) RNA-sequencing analysis of PBMC (n=563 and n=515 respectively) and IHL (n=412 and n=406 respectively; GSE98638). (**m**) Representative flow cytometry plot of gating strategy for intrahepatic leukocytes (IHL) and tumour-infiltrating leukocytes (TIL): lymphocytes, single cells, live cells, $CD45^+$, $CD3^+$, $CD56^-/CD19^-$, $CD4^-/CD8^+$. (**n-s**) IHL from patients with HBV were stimulated with OLP spanning multiple HBV proteins (HBc, HBs, Pol) ±ACAT inhibition (K-604 or DMSO for 16h). Cytokine production of T-cells was detected via flow cytometry. The cytokine production in wells without peptide stimulation was subtracted to determine HBV-specific cytokine production in summary data. (**n**) Summary data for HBV-specific IFNγ production by $CD8^+$IHL ±ACAT inhibition as % of total $CD8^+$ and total $CD45^+$ cells (n=35 OLP pools and 11 donors). (**o**) *De novo* IFNγ production (n=14 OLP pools and 7 donors) by $CD8^+$IHL ±ACAT inhibition. (**p**) HBV-specific TNF production by $CD8^+$IHL ±ACAT inhibition. (**q**) HBV-specific CD107a mobilisation by $CD8^+$IHL ±ACAT inhibition. (**r**) HBV-specific IFNγ production of $CD4^+$IHL ±ACAT inhibition. (**s**) Paired PBMC and IHL stimulated with HBV OLP ±ACAT inhibition (K-604 or DMSO for 16h). Fold change of IFNγ production by $CD4^+$T-cells after stimulation with peptide +ACAT inhibition normalized to peptide +DMSO in patients with detectable pre-existing HBV-specific IFNγ production (n=10 OLP pools and 5 donors). (**t**) Assessment of proliferation by CFSE dilution of aCD3/aCD28 stimulated $CD8^+$T-cells in normal or cholesterol enriched (chol) culture media ±ACAT inhibition (K-604 or DMSO for 4d). (**u**) Representative flow cytometry plot and summary data for CD69 and CD103 co-staining on $CD8^+$IHL ±ACAT inhibition (K-604 or DMSO for 16h; n=35). P values determined by Wilcoxon matched-pairs signed rank test (**b-j, n-u**) or Fisher's exact test (**k,l**).

Supplementary Figure 2

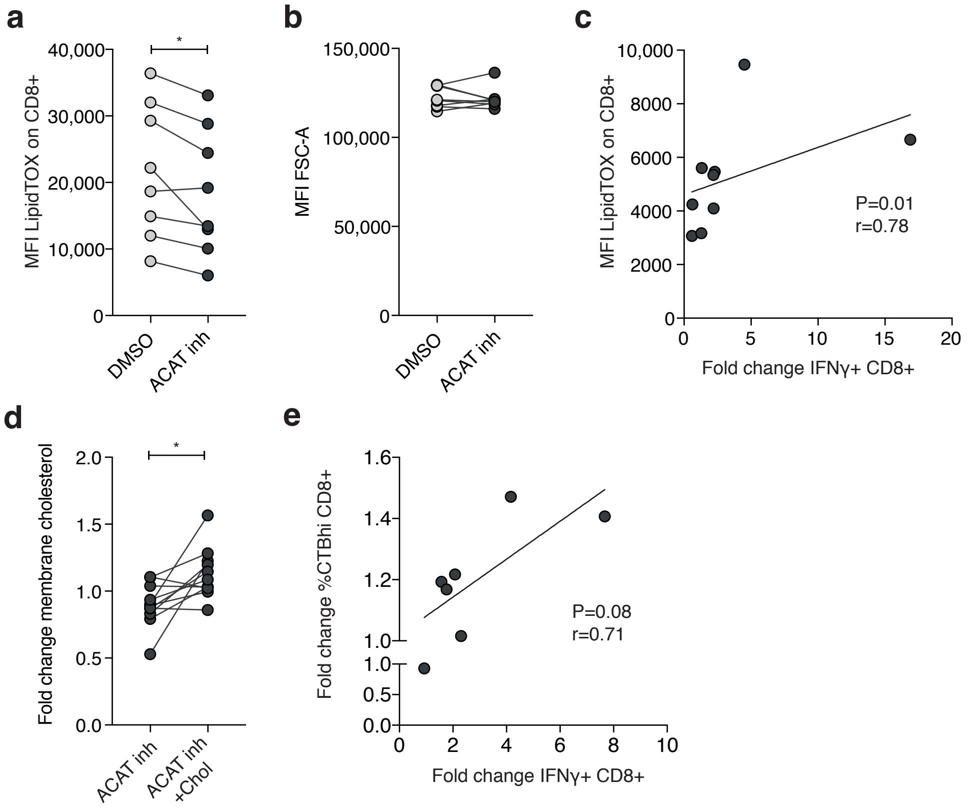

Supplementary Figure 2 ACAT inhibition induces metabolic re-wiring of CD8⁺T-cells.

(**a+b**) Neutral lipid droplets (LipidTOX staining; a) and cell size (determined by FSC-A; b) in unstimulated PBMC ±ACAT inhibition (K-604 or DMSO for 7d; n=8). (**c**) Correlation of ex vivo neutral lipid droplets (LipidTOX staining) in $CD8^+$T-cells from PBMC with fold change of HBV-specific IFNγ production by $CD8^+$T-cells ±ACAT inhibition (Avasimibe or DMSO for 8d; n=9). (**d**) Assessment of free membrane cholesterol by Filipin staining on $CD8^+$T-cells. PBMC cultured in normal or cholesterol enriched (chol) culture media ±ACAT inhibition (K-604 or DMSO for 7d). Fold change of Filipin MFI +ACAT inhibition normalized to DMSO control. (**e**) Correlation of fold change CTB staining and fold change HBV-specific IFNγ production by $CD8^+$T-cells ±ACAT inhibition (Avasimibe or DMSO for 7d; n=7). P values determined by Wilcoxon matched-pairs signed rank test (**a,b,d**) and P and r values by Spearman correlation (**c+e**).

Supplementary Figure 3

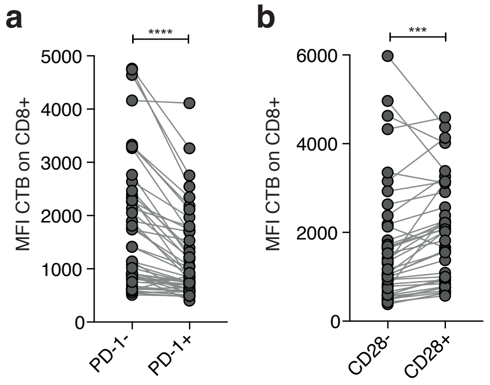

Supplementary Figure 3 Complementary effects of ACAT inhibition and PD-1 blockade.

(**a+b**) Ex vivo staining of $CD8^+$T-cells from PBMC of patients with CHB (**a**) CTB MFI on $PD\text{-}1^{-/+}$ $CD8^+$T-cells (n=46). (**b**) CTB MFI on $CD28^{-/+}$ $CD8^+$T-cells (n=39). P values determined by Wilcoxon matched-pairs signed rank test.

Supplementary Figure 4

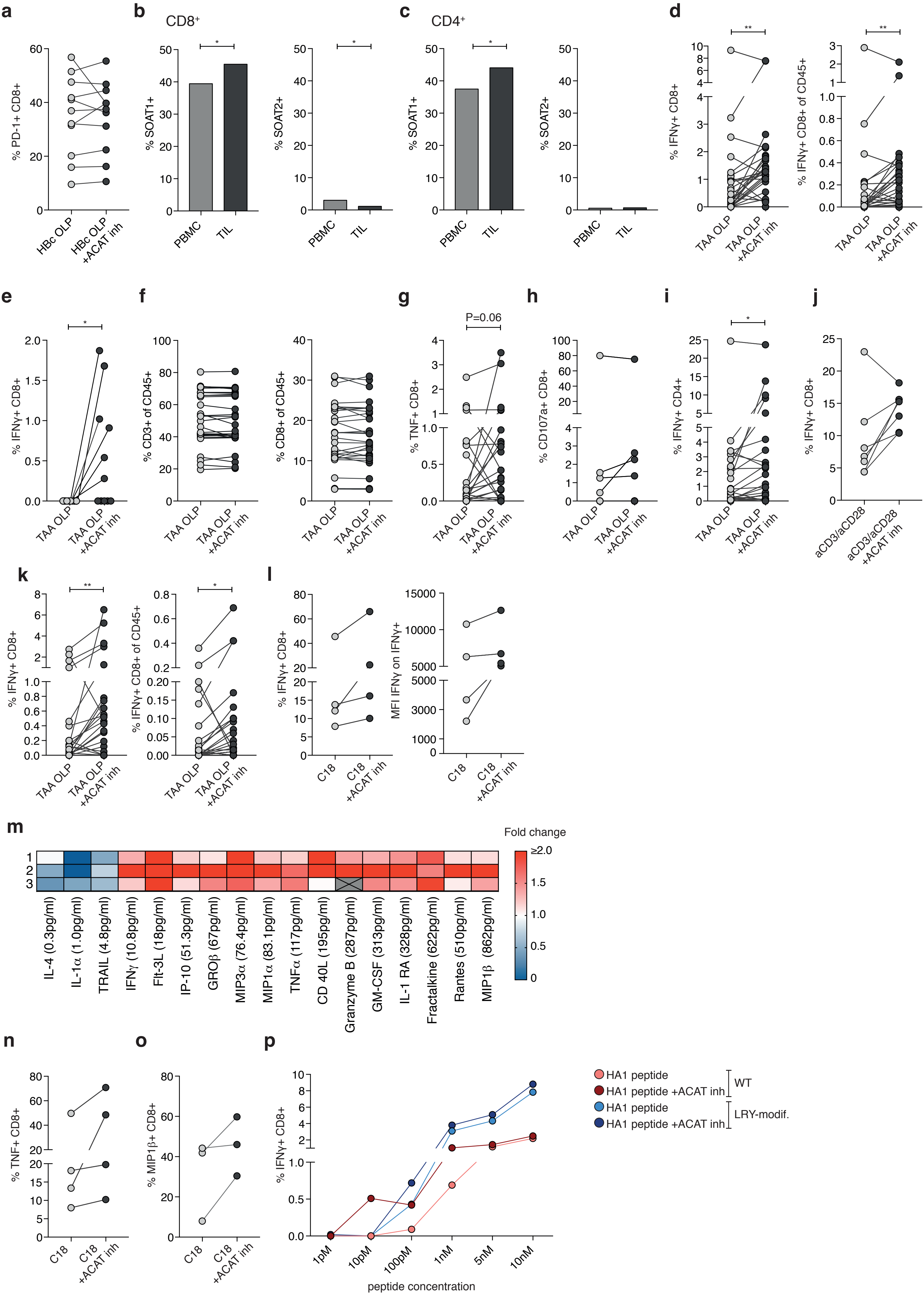

Supplementary Figure 4 ACAT inhibition boosts intratumoural and genetically engineered T-cell responses.

(**a**) PD-1 expression on PBMC $CD8^+$T-cells from patients with CHB stimulated with HBc OLP ±ACAT inhibition (Avasimibe or DMSO for 8d; n=11). (**b+c**) %$CD8^+$ (**b**) and $CD4^+$ (**c**) T-cells with detectable SOAT1 (ACAT1) or SOAT2 (ACAT2) gene transcripts by scRNA-sequencing analysis of PBMC (n=563 and n=515 respectively) and TIL (n=777 and n=606 respectively; GSE98638). (**d-i**) Tumour-infiltrating leukocytes (TIL) from patients with HCC were stimulated with TAA OLP (NY-ESO-1, MAGE-A1, AFP, HBc, HBs, Pol) ±ACAT inhibition (K-604 or DMSO for 16h) and cytokine production of T-cells was detected by flow cytometry. The cytokine production in wells without peptide stimulation was subtracted to determine TAA-specific cytokine production in summary data. (**d**) Summary data for TAA-specific IFNγ production by $CD8^+$TIL ±ACAT inhibition as % of total $CD8^+$ and total $CD45^+$ cells. (**e**) *De novo* IFNγ production of $CD8^+$TIL ±ACAT inhibition (n= 10 OLP pools and 6 donors). (**f**) Frequency of $CD3^+$ and $CD8^+$TIL ±ACAT inhibition. (**g**) TAA-specific TNF production by $CD8^+$TIL ±ACAT inhibition. (**h**) TAA-specific CD107a mobilisation by $CD8^+$TIL ±ACAT inhibition. (**i**) TAA-specific IFNγ production by $CD4^+$TIL ±ACAT inhibition. (**j**) IFNγ production by TIL stimulated with aCD3/aCD28 ±ACAT inhibition (K-604 or DMSO for 16h; n=7). (**k**) Intrahepatic leukocytes (IHL) were isolated from liver tissue surrounding the tumour of patients with HCC and stimulated with TAA OLP (NY-ESO-1, AFP, MAGE-A1) ±ACAT inhibition (K-604 or DMSO for 16h). Detection of IFNγ production by flow cytometry (n= 32 OLP pools from 14 patients). The cytokine production in wells without peptide stimulation was subtracted to determine TAA-specific cytokine production in summary data. (**l-o**) Immune mediator production by HBcAg18-27 -TCR-gene-modified $CD8^+$T-cells cocultured with HepG2 cells pulsed with C18 peptide (25pM for intracellular cytokine staining; 1pM for Luminex) ±ACAT inhibition (Avasimibe or DMSO for 16h). Summary data of 3-4 independent experiments. (**l**) IFNγ production detected by flow cytometry ±ACAT inhibition. (**m**) Immune mediators in supernatant analysed by Luminex assay in 3 independent experiments (row 1-3); Fold change of HBV-specific immune mediator concentration with ACAT inhibition compared to DMSO. Median HBV-specific immune mediator concentration in brackets. Grey box indicates sample with a Granzyme B concentration above the limit of detection. (**n**) TNF production detected by flow cytometry ±ACAT inhibition. (**o**) MIP1β production detected by flow cytometry ±ACAT inhibition. (**p**) IFNγ production of HA1-TCR-gene-modified $CD8^+$T-cells with WT or LRY-modified TCR ±ACAT inhibition (Avasimibe or DMSO for 48h) cocultured with T2 cells pulsed with increasing doses of HA-1 peptide (1pM-10nM). P values determined by Wilcoxon matched-pairs signed rank test (**a, d-l, n,o**) or Fisher's exact test (**b,c**).

Supplementary Figure 5

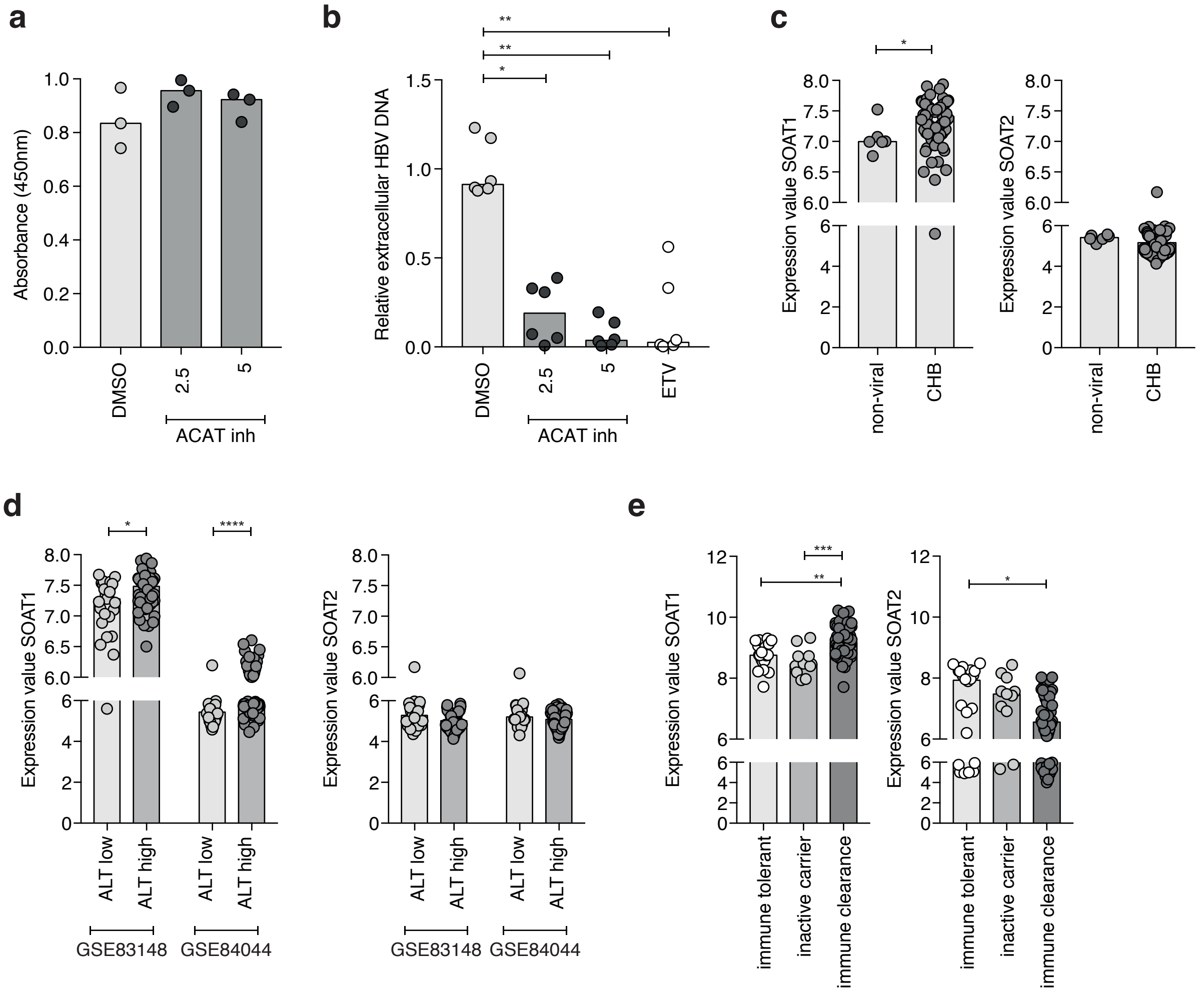

Supplementary Figure 5 ACAT regulates de novo HBV particle genesis.
(**a**) Absorbance (450nm) as indicator of HepG2-NTCP viability ±ACAT inhibition (2.5mg/ml and 5mg/ml Avasimibe or equivalent concentration of DMSO for 6d; n=3). (**b**) Ad-HBV-transduced HepG2 cells were treated with ACAT inhibition (Avasimibe), Entecavir (ETV) or DMSO. Extracellular HBV DNA was measured after 3d in 2 independent experiments in a total of 6 replicates and is expressed as relative to mean of untreated cells. (**c-e**) SOAT (ACAT) gene expression in the liver quantified by microarray. (**c**) Expression values of SOAT1 and SOAT2 in non-viral (n=6) and CHB (n=82); cohort: GSE83148. (**d**) Expression of SOAT1 and SOAT2 in CHB from 2 independent cohorts classified by alanine transferase (ALT) levels into low (≤40IU/L) or high (>40IU/L). GSE83148: low n=25; high n=57. GSE84044 low n=31; high n=62. (**e**) Expression of SOAT1 and SOAT2 classified by disease state into immune tolerant (n=22), inactive carrier (n=11), immune clearance (n=50); cohort: GSE65359. P values determined by Kruskal-Wallis test with Dunn's multiple comparisons test (**a,b,e**) and Mann-Whiney test (**c,d**). Bars indicate median values.

Supplementary Table 1 CHB patient characteristics

Patient	gender	Age [years]	viral load [IU/ml]	HBeAg	ALT [IU/L]	response to ACAT inh
CHB1	f	32	380	negative	29	yes
CHB2	m	44	150	negative	41	yes
CHB3	m	38	BLQ	negative	32	no
CHB4	m	67	250	negative	36	yes
CHB5	m	40	3090	negative	45	no
CHB6	m	25	220	negative	38	yes
CHB7	f	22	31344	negative	37	no
CHB8	m	34	450	negative	39	yes
CHB9	f	42	101	negative	23	yes
CHB10	f	45	1982	negative	35	no
CHB11	m	32	421	negative	58	yes
CHB12	m	44	3300	negative	97	yes
CHB13	m	74	BLQ	negative	24	yes
CHB14	m	32	9300000	positive	98	yes
CHB15	f	39	61	positive	13	yes
CHB16	f	21	7022700	positive	108	yes
CHB17	f	29	80275143	positive	36	no
CHB18	m	28	4360	negative	51	yes
CHB19	f	66	BLQ	negative	66	yes
CHB20	f	32	56	negative	14	yes
CHB21	m	25	2000	negative	26	yes
CHB22	m	47	72172	negative	405	yes
CHB23	f	52	264926	negative	66	no
CHB24	f	47	1101877	positive	42	no
CHB25	m	34	513	negative	24	yes

CHB26	f	31	60	negative	51	no
CHB27	m	32	302	negative	24	yes
CHB28	f	26	634	negative	26	yes
CHB29	m	47	BLQ	negative	35	no
CHB30	m	36	1493	negative	28	yes
CHB31	m	41	408	negative	24	yes
CHB32	f	38	BLQ	negative	21	no
CHB33	f	36	977	negative	21	no
CHB34	f	68	150	negative	22	no

Gender: male (m), female (f); Viral load: below the limit of quantification (BLQ); Alanine aminotransferase (ALT) normal range: 10-50 IU/L; Response to ACAT inhibition defined as increased or *de novo* IFNγ production

Supplementary Table 2: Monoclonal antibodies and fluorescent agents

Antigen	Fluorochrome	Clone	Supplier	Catalogue number	Dilution
**Monoclonal antibodies**					
CD45	BUV805	HI30	BD Bioscience	564914	0.5:100
CD3	BUV395	UCHT1	BD Bioscience	563546	1:100
CD3	BV711	OKT3	Biolegend	317328	1:100
CD3	PE-CF 594	UCHT1	BD Bioscience	562280	1:100
CD8	AlexaFluor700	RPA-T8	Biolegend	301028	1:100
CD8	FITC	RPA-T8	Biolegend	301060	1:100
CD8	BV785	RPA-T8	Biolegend	301046	1:100
CD4	APC-Cy7	RPA-T4	BD Bioscience	557871	1:100
CD4	BUV395	SK3	BD Bioscience	563550	1:100
CD56	PE/Dazzle-594	QA17A16	Biolegend	392410	1:100
CD56	PECy7	HCD56	Biolegend	318317	1:100
CD19	BV786	HIB19	BD Bioscience	740968	1:100
CD69	BV605	FN50	Biolegend	310938	1:100
CD103	BV711	Ber-ACT8	Biolegend	350222	2:100
PD-1	PE	EH12.2H7	Biolegend	329906	2:100

HLA-DR	V500	G46-6	BD Bioscience	561224	1:100
2B4	FITC	C1.7	Biolegend	329505	1:100
CD38	PE-CF594	HIT2	BD Bioscience	562288	0.5:100
CD28	BB700	L293	BD Bioscience	745905	1:100
IFNγ	V450	B27	BD Bioscience	560371	1:100
CD107a	APC	H4A3	BD Bioscience	641581	1.25:100
TNF	FITC	MAb11	BD Bioscience	554512	0.5:100
TNF	APC	MAb11	Biolegend	502912	0.5:100
TNF	APC-Cy7	MAb11	Biolegend	502944	0.5:100
pERK	PerCPCy5.5	6B8B69	Biolegend	369511	2:100
pS6	AlexaFluor647	D57.2.2E	CellSignaling Technology	4851S	2:100
pAKT	PE	M89-61	BD Bioscience	560378	2:100
pAKT	PE-CF594	M89-61	BD Bioscience	563465	2:100
mouse TCRb	APC	H57-597	eBioscience	17-5961-82	1:100
mouse CD19	APC	1D3/CD19	Biolegend	152410	1:100
anti-mouse IgG1	AlexaFluor 546	na	Invitrogen	A-21123	5μg/ml
**Other fluorescent reagents**					
Vybrant CFDA SE Cell Tracer	na	na	Invitrogen	V12883	2.5μM

Kit					
Cholera-toxin B (CTB)	FITC	na	Sigma-Aldrich	C1655-.5MG	25µg/ml
HCS LipidTOX Red Neutral Lipid Stain	na	na	Invitrogen	H34476	1:10.000
Filipin complex from Streptomyces filipinensis	na	na	Sigma-Aldrich	F9765	50µg/ml
LIVE/DEAD Fixable Blue Dead Cell Stain Kit	na	na	Invitrogen	L23105	2:1000
LIVE/DEAD Fixable Near-IR Dead Cell Stain Kit	na	na	Invitrogen	L34976	1.5:1000
CellTracker Deep Red Dye	na	na	Invitrogen	C34565	0.4µM
