# Supplementary material for: Targeting human Acyl-CoA:cholesterol acyltransferase as a dual viral and T cell metabolic checkpoint
Source: Nat Commun. 2021 May 14;12:2814. doi: 10.1038/s41467-021-22967-7 (PMC8121939; doi:10.1038/s41467-021-22967-7)
Supplement: Supplementary file 3 — Reporting Summary [file 41467_2021_22967_MOESM3_ESM.pdf]

nature research

Corresponding author(s): Maini MK
Last updated by author(s): Mar 2, 2021

Reporting Summary

Nature Research wishes to improve the reproducibility of the work that we publish. This form provides structure for consistency and transparency in reporting. For further information on Nature Research policies, see Authors & Referees and the Editorial Policy Checklist .

Statistics

For all statistical analyses, confirm that the following items are present in the figure legend, table legend, main text, or Methods section.

n/a	Confirmed	
[ ]	[x]	The exact sample size (*n*) for each experimental group/condition, given as a discrete number and unit of measurement
[ ]	[x]	A statement on whether measurements were taken from distinct samples or whether the same sample was measured repeatedly
[ ]	[x]	The statistical test(s) used AND whether they are one- or two-sided<br>*Only common tests should be described solely by name; describe more complex techniques in the Methods section.*
[x]	[ ]	A description of all covariates tested
[ ]	[x]	A description of any assumptions or corrections, such as tests of normality and adjustment for multiple comparisons
[ ]	[x]	A full description of the statistical parameters including central tendency (e.g. means) or other basic estimates (e.g. regression coefficient) AND variation (e.g. standard deviation) or associated estimates of uncertainty (e.g. confidence intervals)
[ ]	[x]	For null hypothesis testing, the test statistic (e.g. *F*, *t*, *r*) with confidence intervals, effect sizes, degrees of freedom and *P* value noted<br>*Give P values as exact values whenever suitable.*
[x]	[ ]	For Bayesian analysis, information on the choice of priors and Markov chain Monte Carlo settings
[x]	[ ]	For hierarchical and complex designs, identification of the appropriate level for tests and full reporting of outcomes
[x]	[ ]	Estimates of effect sizes (e.g. Cohen's *d*, Pearson's *r*), indicating how they were calculated

Our web collection on statistics for biologists contains articles on many of the points above.

Software and code

Policy information about availability of computer code

Data collection	Software used for data acquisition:FACSDIVA v8.0.0.1; softWoRx 5.0; Seahorse Wave Desktop Software
Data analysis	Software used for data/statistical analysis: FlowJo v.10; Prism 7.0; Excel v.16.16.09;

For manuscripts utilizing custom algorithms or software that are central to the research but not yet described in published literature, software must be made available to editors/reviewers. We strongly encourage code deposition in a community repository (e.g. GitHub). See the Nature Research guidelines for submitting code & software for further information.

Data

Policy information about availability of data

All manuscripts must include a data availability statement. This statement should provide the following information, where applicable:

- Accession codes, unique identifiers, or web links for publicly available datasets
- A list of figures that have associated raw data
- A description of any restrictions on data availability

The raw data that support the findings of this study are available from the corresponding author upon reasonable request.

Field-specific reporting

Please select the one below that is the best fit for your research. If you are not sure, read the appropriate sections before making your selection.

[x] Life sciences [ ] Behavioural & social sciences [ ] Ecological, evolutionary & environmental sciences

For a reference copy of the document with all sections, see nature.com/documents/nr-reporting-summary-flat.pdf

Life sciences study design

All studies must disclose on these points even when the disclosure is negative.

Sample size	No statistical methods were used to predetermine sample size. The sample size was selected based on published studies in the field (Schurich Cell Rep. 2016; Schurich PLoS Pathog. 2013; Nebbia PLoS One 2012; Boni J Virol 2007; Bengsch J Hepatol 2014) and on available patient tissue samples.
Data exclusions	All tissue samples with sufficient numbers of isolated viable lymphocytes were included in this study.
Replication	For phenotypic and functional assessment of human T-cells by flow cytometry or bioenergetic measurements, experiments were performed with samples from at least 6 donors (n numbers are stated in figure legends). Experimental variation between donors has been depicted graphically with all data points displayed in each figure. The experimental techniques used (e.g. leukocyte isolation, monoclonal antibody staining, flow cytometry, bioenergetic measurements, data analysis) were repeatedly performed on independent days.<br>Experiments to measure the antiviral effect were performed in 4-6 replicates as stated in figure legend (Fig.5).
Randomization	No randomization was performed. All tissue donors were pseudonymised.
Blinding	During the analysis the investigators were blinded to all clinical patient data. There were no other experiments amenable to blinding.

Reporting for specific materials, systems and methods

We require information from authors about some types of materials, experimental systems and methods used in many studies. Here, indicate whether each material, system or method listed is relevant to your study. If you are not sure if a list item applies to your research, read the appropriate section before selecting a response.

Materials & experimental systems

n/a	Involved in the study	
☐	☒	Antibodies
☐	☒	Eukaryotic cell lines
☒	☐	Palaeontology
☒	☐	Animals and other organisms
☐	☒	Human research participants
☐	☒	Clinical data

Methods

n/a	Involved in the study	
☒	☐	ChIP-seq
☐	☒	Flow cytometry
☒	☐	MRI-based neuroimaging

Antibodies

Antibodies used	Detailed information regarding all antibodies and other fluorescent agents used in this study are listed in Supp.Table 2
Validation	All antibodies were purchased from well established manufacturers and would have been validated by the vendor for species and target. Clones and catalogue numbers for each antibody have been included for cross-referencing of manufacturing company specification/validation processes in Supp. Table 2.<br>We further validated antibodies by titration to optimal concentrations and by using positive controls where possible (e.g. using populations known to express a certain marker or by polyclonal stimulation). Antibody concentrations are stated in Supp Table 2.

Eukaryotic cell lines

Policy information about cell lines

Cell line source(s)	HepG2-NTCP: gifted by Stephan Urban, Heidelberg, who established this cell line (Ni Gastroenterology 2014).<br>T2: purchased from ATCC (T2 (174 x CEM.T2) (ATCC® CRL-1992™))<br>Phoenix-AMPHO: purchased from ATCC (Phoenix-AMPHO (ATCC® CRL-3213™))<br>HepG2: purchased from ATCC (Hep G2 [HEPG2] (ATCC® HB-8065™))
Authentication	HepG2-NTCP: cell line established by Stephan Urban (Ni Gastroenterology 2014). NTCP expression was confirmed by our lab.<br>T2, Phoenix-AMPHO, HepG2: Cell lines were commercially purchased from a well established company and would have been validated by the company.<br>We did not perform further cell line authentication.
Mycoplasma contamination	All cell lines used in this study are regularly tested negative for mycoplasma contamination by PCR (EZ-PCR Mycoplasm Kit, Biological Industries).

Commonly misidentified lines (See ICLAC register)	No commonly misidentified cell lines were used in this study.

Human research participants

Policy information about studies involving human research participants

Population characteristics	Each study participant provided written informed consent prior to inclusion in the study, with the storage of any samples/clinical information complying with the requirements of the Human Tissue Act 2004. Peripheral blood samples were taken from healthy control individuals, patients with CHB (detailed characteristics in Supp Table 1) and patients with HCC. Resected liver tissue from HBV-infected livers, tumour-surrounding non-tumour liver tissue, liver tumour resections and paired blood samples were obtained through the Tissue Access for Patient Benefit (TapB) scheme at The Royal Free Hospital.
Recruitment	Peripheral blood samples were taken from healthy control individuals (recruited at UCL), patients with CHB (recruited at the Royal Free Hospital and at the Mortimer Market Centre, London) and patients with HCC (recruited at the Royal Free Hospital, London). Resected liver tissue from HBV-infected livers, tumour-surrounding non-tumour liver tissue, liver tumour resections and paired blood samples were obtained through the Tissue Access for Patient Benefit (TapB) scheme at The Royal Free Hospital. Each participant gave written informed consent before inclusion.
Ethics oversight	This study was approved by the local ethics boards (Wales Research Ethics Committee 4 and UCL Biobank Ethical Review Committee: Research Ethics Committee reference number 16/WA/0289; Brighton and Sussex: Research Ethics Committee reference number 11/LO/0421)

Note that full information on the approval of the study protocol must also be provided in the manuscript.

Clinical data

Policy information about clinical studies

All manuscripts should comply with the ICMJEguidelines for publication of clinical research and a completedCONSORT checklist must be included with all submissions.

Clinical trial registration	Not applicable
Study protocol	Not applicable
Data collection	Clinical data (e.g. HBeAg status, HBV DNA level, ALT) was collected at the time point of tissue sample donation.
Outcomes	Not applicable

Flow Cytometry

Plots

Confirm that:

- [x] The axis labels state the marker and fluorochrome used (e.g. CD4-FITC).
- [x] The axis scales are clearly visible. Include numbers along axes only for bottom left plot of group (a 'group' is an analysis of identical markers).
- [x] All plots are contour plots with outliers or pseudocolor plots.
- [x] A numerical value for number of cells or percentage (with statistics) is provided.

Methodology

Sample preparation	PBMC were isolated from heparinized blood by density centrifugation with Ficoll-Hypaque Plus (GE Healthcare) or Pancoll (Pan Biotech). PBMC were used fresh or were cryopreserved in 10% DMSO (Sigma-Aldrich) prior to further use.<br><br>To isolate IHL and TIL, liver/tumour tissue was cut into small pieces and incubated for 30min at 37C in 0.01% collagenase IV (Invitrogen) and 0.001% DNAse I (Sigma-Aldrich), followed by further mechanical disruption via GentleMACS (Miltenyi Biotech), filtration through a 70um cell strainer (BD Bioscience), removal of parenchymal cells on a 30% Percoll gradient (GE Healthcare) and lymphocyte isolation via density centrifugation as described above. IHL and TIL were used immediately after isolation.
Instrument	BD Bioscience Fortessa-X20
Software	FlowJo v.10 (TreeStar/BD Bioscience)
Cell population abundance	Sample purity, where appropriate, was confirmed by flow cytometry to confirm purities >98%.
Gating strategy	The gating strategy used for PBMC is shown in Supp. Fig1a: cells were gated on their size and granularity to identify lymphocytes, single cells, live cells, CD3+, CD4-/CD8+ or CD4+/CD8-. The gating strategy used for IHL and TIL is shown in Supp. Fig1m: cells were gated on their size and granularity to identify lymphocytes and exclude residual hepatocytes/tumour cells, single cells, live

cells, CD45+, CD3+, CD56-/CD19-, CD4-/CD8+. or CD4+/CD8-

☒ Tick this box to confirm that a figure exemplifying the gating strategy is provided in the Supplementary Information.
